# Supplementary material for: LAFA: a framework for reproducible longitudinal assessment of protein function annotation models
Source: Bioinform Adv. 2026 Aug 7;6(1):vbag221. doi: 10.1093/bioadv/vbag221 (PMC13501529; doi:10.1093/bioadv/vbag221)
Supplement: Supplementary file 1 [file vbag221_supplementary_data.pdf]

Supplementary Materials to LAFA: A Framework for the Longitudinal Assessment of Protein Function Annotation Models

An Phan, Yanli Wang, Frimpong Boadu,
Jianlin Cheng, Predrag Radivojac and Iddo Friedberg

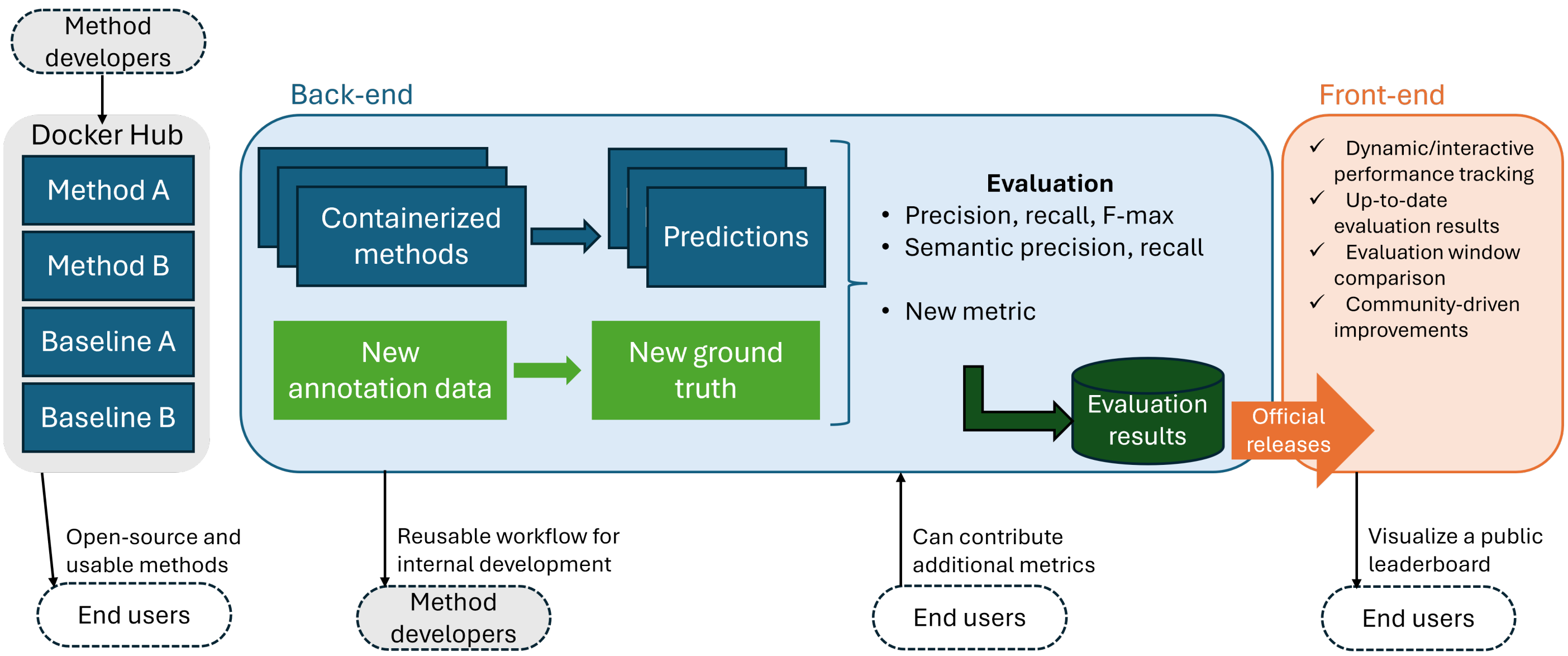

Figure S1: The back-end and front-end infrastructure of LAFA.

1 Baseline methods

In previous rounds of CAFA and currently for LAFA, we have used four baseline methods to provide a reference point in assess protein function prediction methods. The rationale for using the four baselines is as follows:

- Non-Experimental GOA baseline: This baseline evaluates how accurately the GO annotations supported by non-experimental evidence codes (including electronic, computational, and phylogenetically inferred evidence codes) predict future experimentally validated functions. It is a simple baseline that reflects the non-experimental annotations collectively, which often get validated experimentally in the future. In addition, the non-experimental GOA baseline has been shown to perform relatively well in CAFA5 [1].
- Naive baseline: This baseline assigns every protein with the prior probability of each GO term in the database of experimentally annotated proteins. In other words, the Naive baseline captures how often each GO term is assigned to proteins regardless of sequence information. It is a traditional CAFA baseline that has been used since CAFA1 [2].
- BLAST baseline: BLAST remains a standard and widely used method for annotation transfer from experimentally annotated proteins based on homology information. Similar to the Naive baseline, BLAST is also a traditional CAFA baseline that has been used since CAFA1 [2].
- Embedding similarity baseline: This is a protein-embedding-based baseline built from the ProtT5 protein language model [3]. It was introduced in CAFA5 to reflect the increasing adoption of protein language models in state-of-

the-art methods [1]. Competitive methods should demonstrate improved performance over the simple Euclidean distance-based annotation transfer strategy used in the embedding similarity baseline.

2 Evaluation metrics

At each new UniProt-GOA release t_1, we collect the ground truth set as the experimentally validated annotations that were added between prediction time t_0 and evaluation time t_1, in all aspects of GO: molecular function, biological process, and cellular component. A protein is evaluated only on annotations that were not present for that protein at t_0. The time-delayed design follows the CAFA evaluation principle as follows.

First, proteins are classified into three benchmark subsets according to their annotation status at t_0: no-knowledge, limited-knowledge, or partial-knowledge [4]. The **no-knowledge** subset contains proteins that are experimentally unannotated at t_0 and have acquired new GO annotations by t_1. For proteins that have been experimentally annotated at t_0, they are assigned to the **limited-knowledge** subset if they gain annotations in a previously unannotated GO aspect, and/or to the **partial-knowledge** subset if they gain annotations in the previously annotated GO aspect(s). Thus, no-knowledge proteins are distinct from the limited- and partial-knowledge cases.

Second, predictions are evaluated after propagation according to the GO true-path rule, so that predicted terms are interpreted consistently within the ontology hierarchy. For each method, prediction files in protein-term-score format are compared with the corresponding protein-term ground truth over score thresholds from 0 to 1 in increments of 0.01. This produces threshold-dependent precision, recall, and F_1 scores. In addition to the unweighted measures, CAFA-style evaluation also uses information-theoretic weighting to emphasize the correct prediction of more specific terms [5, 6, 7]. Each GO term is assigned a weight based on its information accretion, so rarer and more informative terms contribute more strongly than broad, high-level terms. The rationale for using weighted precision and recall is to distinguish predictors that recover specific biological information from those that mainly recover generic ancestor terms, which are easier to predict and less informative. The root terms appear in every protein's annotation and thus, their weights are 0.

Finally, precision, recall, and the maximum F_1 score are computed separately for each benchmark subset within each GO aspect. Currently, the metrics displayed in LAFA are micro-averaged weighted metrics, meaning that predictions are pooled across proteins before calculating summary statistics. All evaluations are run with the CAFA-evaluator package [8], and the partial-knowledge extension is available at `https://github.com/claradepaolis/CAFA-evaluator-PK`.

3 Case study: Obsolete GO terms

Since methods cannot foresee the newly obsolete GO terms during the annotation accumulation window, some predictions generated t_0 could become obsolete predictions at t_1.

As an example, we selected the time window "Sep_2025_Mar_2026" to collect some statistics. In the table, we count the number of obsolete GO terms in Sep_2025 and Mar_2026 timepoints, and the percentage of predictions from each method that were affected by these obsolete GO terms. The obsoletion problem occurs with all the baseline methods (even though they have 0 obsolete predictions at Sep_2025), and is worse for the three submitted methods (DeepGOPlus, TransFew, FunBind) because these methods do not take the Sep_2025 GO graph as an input. The newly obsolete GO terms in Mar_2026 only affected around 1% (numbers in red) of predictions from four baseline and three submitted methods. Most of the obsolete predictions of FunBind and TransFew were already obsolete in Sep_2025 because these methods used outdated training data.

	Sep_2025 GO graph (release 2025-07-22)	**Newly obsolete in Mar_2026 GO graph (release 2026-01-23)**
Obsolete GO terms (total terms in brackets)	8,259 (total 48,165 terms)	1,256 (total 48,251 terms)
	Obsolete predictions percentage (based on Sep_2025 graph)	**Newly obsolete predictions percentage (based on Mar_2026 graph)**
Baseline: BLAST	0%	0.88%
Baseline: Naive	0%	0.14%
Baseline: GOA Non-exp	0%	0.33%
Baseline: Embedding	0%	0.33%
DeepGOPlus	0.25%	0.81% (1.05% in total)
FunBind	7.08%	1.10% (8.18% in total)
TransFew	7.73%	1.06% (8.79% in total)

Obsolete leaf GO terms are not included in the CAFA/LAFA evaluation, however, their ancestors are still included in the evaluation because we perform propagation before evaluation. Therefore, we further checked whether the newly obsolete GO predictions are mapped to valid ancestral GO terms. We found that all affected GO term-protein pairs from all methods have valid ancestral GO terms in Mar_2026, meaning that these predictions were not removed from the evaluation just because they are made to obsolete GO terms.

4 Example use cases

Here, we elaborate on the use of LAFA's main workflows to generate all required data for each time point and time window. The examples below have been run for each time point and time window to get the results displayed on LAFA currently. LAFA's back-end pipeline has been documented in detail at `https://github.com/anphan0828/CAFA_forever/blob/main/README_backend.md`

4.1 Time point build

The time point build workflow is responsible for creating a LAFA snapshot for any time point, including GO graph, filtered experimental annotations, training set of proteins, information accretion values (for weighted precision-recall). The resulting directory serves as the reusable foundation for downstream prediction and evaluation.

The command:

```
nextflow run main.nf --mode timepoint_build
    --timepoint_id Sep_2025
    --timepoint_root data/time_points/Sep_2025
```

creates a folder using data associated with the release ID "Sep_2025" which is then published on HuggingFace repository. After these data have been prepared, predictions of all proteins in the `test set` (all SwissProt protein sequences available at the time) are produced for all methods.

4.2 Time window evaluation

The time window evaluation is the core workflow that generates all components needed for a LAFA evaluation between any two time points. This workflow identifies shared protein targets (with similar amino acid sequences), classifies proteins into no-knowledge, limited-knowledge, and partial-knowledge subsets, and runs the `CAFA-evaluator` package across the prediction set associated with the earlier time point [8]. The command:

```
nextflow run main.nf --mode evaluate_window
  --t0_id Sep_2025
  --t1_id Nov_2025
  --t0_root data/time_points/Sep_2025
  --t1_root data/time_points/Nov_2025
  --publish_root data/releases
```

produces ground truth files and evaluation results for methods whose predictions existed at t_0.

In this study, predictions for proteins in the temporary small `ground truth target set` are collected and immediately evaluated as follows:

```
nextflow run main.nf --mode evaluate_window
  --t0_id Sep_2025   --t1_id Mar_2026
  --t0_root data/time_points/Sep_2025
  --t1_root data/time_points/Mar_2026
  --publish_root data/releases
  --window_prediction_mode existing
  --window_predictions_dir
  data/time_points/Sep_2025/predictions_by_window/Sep_2025_Mar_2026
```

The additional arguments `--window_prediction_mode` and `--window_predictions_dir` offer the choice to (1) evaluate all predictions in a pre-existing directory (when `--window_prediction_mode` is 'existing') or (2) create predictions for existing methods and then evaluate (when `--window_prediction_mode` is 'generate'). When these two additional arguments are provided, the workflow will first generate the `ground truth target set` and then pass this set into method containers. The subsequent evaluation step also performs on this smaller set of proteins that we know are included in the ground truth. This makes it extremely convenient for LAFA to cut down on run time and use a smaller set of predictions to perform evaluation.

References

[1] M Clara De Paolis Kaluza, Rashika Ramola, Parnal Joshi, Damiano Piovesan, Walter Reade, Sandra Orchard, Maria J Martin, Alex Ignatchenko, Kaggle Competition Participants, Burkhard Rost, et al. Advances in protein function prediction from the fifth CAFA challenge. *bioRxiv*, pages 2026–04, 2026.

[2] Predrag Radivojac, Wyatt T Clark, Tal Ronnen Oron, Alexandra M Schnoes, Tobias Wittkop, Artem Sokolov, Kiley Graim, Christopher Funk, Karin Verspoor, Asa Ben-Hur, et al. A large-scale evaluation of computational protein function prediction. *Nature Methods*, 10(3):221–227, 2013.

[3] Ahmed Elnaggar, Michael Heinzinger, Christian Dallago, Ghalia Rehawi, Yu Wang, Llion Jones, Tom Gibbs, Tamas Feher, Christoph Angerer, Martin Steinegger, Debsindhu Bhowmik, and Burkhard Rost. ProtTrans: Toward Understanding the Language of Life Through Self-Supervised Learning. *IEEE Transactions on Pattern Analysis and Machine Intelligence*, 44(10):7112–7127, 2022.

[4] Yuxiang Jiang, Tal Ronnen Oron, Wyatt T Clark, Asma R Bankapur, Daniel D'Andrea, Rosalba Lepore, Christopher S Funk, Indika Kahanda, Karin M Verspoor, Asa Ben-Hur, et al. An expanded evaluation of protein function prediction methods shows an improvement in accuracy. *Genome Biology*, 17(1):184, 2016.

[5] Wyatt T Clark and Predrag Radivojac. Information-theoretic evaluation of predicted ontological annotations. *Bioinformatics*, 29(13):i53–i61, 2013.

[6] Iddo Friedberg, Predrag Radivojac, Clara De Paolis, Damiano Piovesan, Parnal Joshi, Walter Reade, and Addison Howard. CAFA 5 protein function prediction. `https://kaggle.com/competitions/cafa-5-protein-function-prediction`, 2023. Kaggle.

[7] Iddo Friedberg, Predrag Radivojac, Paul D Thomas, An Phan, M. Clara De Paolis Kaluza, Damiano Piovesan, Parnal Joshi, Chris Mungall, Martyna Plomecka, Walter Reade, and María Cruz. CAFA 6 protein function prediction. `https://kaggle.com/competitions/cafa-6-protein-function-prediction`, 2025. Kaggle.

[8] Damiano Piovesan, Davide Zago, Parnal Joshi, M Clara De Paolis Kaluza, Mahta Mehdiabadi, Rashika Ramola, Alexander Miguel Monzon, Walter Reade, Iddo Friedberg, Predrag Radivojac, et al. CAFA-evaluator: a python tool for benchmarking ontological classification methods. *Bioinformatics Advances*, 4(1):vbae043, 2024.
